# Pharmacovigilance of herbal medicines: Current state and future directions

**DOI:** 10.4103/0973-1296.75905

**Published:** 2011

**Authors:** Sandeep Shetti, C. Dinesh Kumar, Neeraj Kumar Sriwastava, Indra Prakash Sharma

**Affiliations:** *Department of Pharmacognosy, Manipal College of Pharmaceutical Sciences, Manipal - 576 104, India*; 1 *Masterskill University College of Health Sciences, Cheras - 43200, Kuala Lumpur, Malaysia*

**Keywords:** Guidelines, herbal medicines, pharmacovigilance, regulatory

## Abstract

Currently, a majority of the adverse events related to the use of herbal products and herbal medicines that are reported are attributable either to poor product quality or to improper use. Inadequate regulatory measures, weak quality control systems, and largely uncontrolled distribution channels (including mail order and Internet sales) may have been contributing to the occurrence of such events. In order to expand the knowledge about genuine adverse reactions to herbal medicines, and to avoid wasting scarce resources for identifying and analyzing adverse events, events resulting from such situations will need to be reduced or eliminated. Member States of the World Health Organization (WHO) are therefore encouraged to strengthen national regulation, registration and quality assurance and control of herbal medicines. In addition, the national health authorities should give greater attention to consumer education and to qualified practice in the provision of herbal medicines.

## INTRODUCTION

The WHO has welcomed the active participation of drug regulatory authorities and national pharmacovigilance centers, among others, in the development of these guidelines. This has provided a useful starting point for strengthening communication between these authorities, which will be needed to ensure progress toward the common goal—the safety of herbal medicines. The recommended approach is to include herbal medicines in the existing national pharmacovigilance systems or, where such systems have not yet been developed, to establish comprehensive national pharmacovigilance systems, which incorporate coverage of herbal medicines.[1] The guidelines therefore identify the particular challenges posed in monitoring the safety of herbal medicines effectively and propose approaches for overcoming them. Special attention is also given to the reporting system for adverse reactions to herbal medicines, and to the analysis of the causes of the reported adverse reactions.[2]

Safety is a fundamental principle in the provision of herbal medicines and herbal products for health care, and a critical component of quality control. These guidelines provide practical technical guidance for monitoring the safety of herbal medicines within the pharmacovigilance systems. The safety monitoring of herbal medicines is compared and contrasted with that of other medicines currently undertaken in the context of the WHO International Drug Monitoring Program. Although there are regulatory and cultural differences in the preparation and use of different types of medicines, they are all equally important from a pharmacovigilance perspective.

The guidelines were developed with the view that, within the current pharmacovigilance systems, monitoring of the safety of medicines should be enhanced and broadened in ways that will allow the successful monitoring of herbal medicines. The inclusion of herbal medicines in pharmacovigilance systems is becoming increasingly important given the growing use of herbal products and herbal medicines globally. For example, in the United States of America, some US$ 17 billion were spent by more than 158 million Americans in 2000. Furthermore, a recent study indicated that more than 70% of the German population reported using “natural medicines” and that, for most of them, herbal medicinal products were the first choice in the treatment of minor diseases or disorders. The worldwide consumption of herbal medicines today is enormous, so that, in terms of population exposure alone, it is essential to identify the risks associated with their use. Safety of herbal medicines is therefore an important public health issue. Herbal medicines are frequently used in conjunction with other medicines, and it is essential to understand the consequences of such combined use and monitor whether any adverse effects are arising. This can be achieved most readily within the existing pharmacovigilance systems. To handle herbal medicines and, in particular, to analyze the causes of adverse events, the national pharmacovigilance centers (or equivalent institutions) will need to acquire specific technical expertise. This will include trained personnel in the relevant technical areas and facilities to analyze the products concerned, for which there is often insufficient information and lack of access to reliable information support. Many countries currently lack this expertise and, in particular, access to suitable analytic laboratories. Member States have therefore recommended the establishment of regional laboratories specializing in the analysis of herbal products. The WHO encourages Member States to explore the feasibility of this proposal.

Despite the growing interest in the safety of herbal medicines, national surveillance systems to monitor and evaluate adverse reactions associated with herbal medicines are rare, even among the more than 70 Member States participating in the WHO International Drug Monitoring Program. Moreover, there is a lack of effective communication on this subject at all levels, from international to local. A recent WHO survey showed that around 90 countries, less than half of WHO’s Member States, currently regulate herbal medicines, and an even smaller proportion has systems in place for the regulation/qualification of providers of herbal medicines. Moreover, there are disparities in regulation between different countries, and this has serious implications for international access to and distribution of such products.[3]

National pharmacovigilance systems should be closely linked to the national drug regulatory systems. To function properly, a national safety monitoring program for herbal medicines should be operated alongside an effective national drug regulatory system with the will and the potential to react to signals emanating from reports of adverse effects of herbal medicines and to take proper regulatory measures. At the national level, the capacity for reporting adverse events on herbal medicines, analyzing their causes and learning from past experience is seriously hampered in many Member States by the lack of methodologic uniformity in identification and measurement, the lack of information on adverse effects of herbal medicines, inadequate reporting schemes, fear of professional liability, and inadequate information systems relating to the use of herbal medicines. Current knowledge of the epidemiology of adverse reactions to herbal medicines, such as frequency of occurrence and causes, is very limited.[4]

The WHO has taken the lead in tackling the need for drug safety monitoring since 1970 (resolution WHA23.13 on international monitoring of adverse reactions to drugs, 1970). The WHO International Drug Monitoring Program, together with the WHO Collaborating Centre in Sweden, the Uppsala Monitoring Centre (UMC), has instituted a coherent program of action for pharmacovigilance, which includes the establishment of a program for exchange of safety information, maintenance of the global WHO database of adverse drug reaction (ADR) reports (hereafter referred to as the global WHO database), and the provision of numerous guidelines on monitoring drug safety. It also seeks to bridge the gap between the industry and the regulatory authorities. As an immediate response to the need for pharmacovigilance for herbal medicines, the WHO has increased its efforts to promote their safety monitoring within the context of the WHO International Drug Monitoring Program.

## PHARMACOVIGILANCE AND THE WHO INTERNATIONAL DRUG MONITORING PROGRAM

Pharmacovigilance is the science and activities relating to the detection, assessment, understanding, and prevention of the adverse effects of drugs or any other possible drug-related problems.[5]

Recently, its concerns have been widened to include the following:

♦ herbals
♦ traditional and complementary medicines
♦ blood products
♦ biologicals
♦ medical devices
♦ vaccines.

The specific aims of Pharmacovigilance are to:

♦ improve patient care and safety in relation to the use of medicines and all medical and paramedical interventions
♦ improve public health and safety in relation to the use of medicines
♦ contribute to the assessment of benefit, harm, effectiveness, and risk of medicines, encouraging their safe, rational, and more effective (including cost-effective) use
♦ promote understanding, education, and clinical training in pharmacovigilance and its effective communication to the public.

### The WHO international drug monitoring program[6]

Under the WHO International Drug Monitoring Program, national pharmacovigilance centers designated by the competent health authorities are responsible for the collection, processing, and evaluation of case reports of suspected adverse reactions supplied by health care professionals (mainly spontaneous reporting by physicians of reactions associated with the use of prescribed medicines). The Program is described in two publications: Safety monitoring of medicinal products: guidelines for setting up and running a pharmacovigilance center,[1] chapters 7 and 8; and The importance of pharmacovigilance: safety monitoring of medicinal products,[5] especially chapters 3 and 4.

The Program currently comprises a network of more than 70 national pharmacovigilance centers that operate independently, but whose functions are coordinated and facilitated by the WHO and the UMC. The UMC manages the global WHO database to which all case reports received by the national pharmacovigilance centers are sent. The UMC uses the global WHO database to identify/detect signals of new adverse reactions from the cumulative data and to communicate risk assessments back to the national pharmacovigilance centers and to others concerned with drug safety.[7]

## CHALLENGES IN MONITORING THE SAFETY OF HERBAL MEDICINES

### Regulation

National regulation and registration of herbal medicines vary from country to country. Where herbal medicines are regulated, they may be categorized as either prescription or non-prescription medicines. Herbal products may also be categorized other than as medicines. Moreover, the regulatory status of a particular herbal product may differ in different countries. The national regulatory framework usually also includes involved qualified providers and distributors of the respective substances. Regulatory status consequently determines the access to or distribution route of these products.[8]

#### Quality assurance and control

Quality assurance and control measures, such as national quality specification and standards for herbal materials, good manufacturing practices (GMP) for herbal medicines, labeling, and licensing schemes for manufacturing, imports and marketing, should be in place in every country where herbal medicines are regulated.[9] These measures are vital for ensuring the safety and efficacy of herbal medicines. Weak regulation and quality control may result in a high incidence of adverse reactions attributable to poor quality of herbal medicines, in particular, resulting from adulteration with undeclared potent substances and/or contamination with potentially hazardous substances and residues.

The requirements and methods for quality control of finished herbal products, particularly for mixture herbal products, are far more complex than for other pharmaceuticals. The quality of such products is influenced by the quality of the raw material used. Good agricultural and good collection practices for medicinal plants, including plant selection and cultivation, are therefore important measures.

#### Safety monitoring of herbal medicines

The most common sources of information on adverse events and reactions to medicines are clinical trials and spontaneous reports (voluntary, unsolicited communications on marketed medicinal products). The latter ordinarily far exceed the former in numbers and type, especially serious reports, over the lifetime of a product. In some countries, adverse reaction reporting by physicians is mandatory; such reports are regarded as spontaneous.

In many countries, providers of herbal medicines other than physicians, dentists, pharmacists, and nurses are excluded from reporting systems. If adequate coverage of herbal medicines is to be achieved, national reporting schemes should be developed to include all providers of herbal medicines (both prescribers and dispensers), and providers of traditional, complementary, and alternative medicine, according to national circumstances.

A substantial proportion of herbal medicines are nonprescription medicines, and many come directly into this category without prior postmarketing safety monitoring as prescription medicines. It is therefore most important to take measures to strengthen pharmacovigilance activity in the nonprescription medicines setting. Community pharmacists and nurses can play a particularly useful role in monitoring the safety of nonprescription medicines, although many such products are sold outside pharmacies. The involvement of consumers in the use of herbal medicines and herbal products in health care, and their concern regarding possible adverse effects should be valued positively. Consumer reports on adverse reactions should be accepted as an important source of information, which can contribute to the identification of signals for unknown effects of herbal medicines.

#### Recording and coding the identity of herbal medicines

Use of a standardized classification and identification for transmitting reports to the UMC is desirable. Coding of adverse events/adverse reactions to herbal medicines should be compatible with that for other medicines. The UMC therefore proposes the use of the WHO Drug Dictionary (WHO-DD), as it has been developed to store structured, classified information on the names of herbal products and their ingredients in the same way as similar information on other medicines. For the therapeutic classification of herbal products, the UMC proposes the herbal anatomic–therapeutic–chemical (HATC) classification, which is structurally equivalent to the anatomic–therapeutic–chemical (ATC) classification used for chemical substances in other medicines. HATC is being implemented within the WHO-DD structure as part of the global WHO database. A combination of the use of the HATC classification and the expanded global WHO database structure can manage all levels of data input, however imprecise. In addition, the UMC also proposes a system checklist for cross-referencing of botanical and vernacular names used as names of ingredients. The UMC suggests that the WHO-DD, the HATC classification and the checklist should prove useful tools for the national pharmacovigilance centers when asking questions of the reporter to increase the clarity and accuracy of reports.

Herbal medicines usually contain multiple ingredients and it is not always possible to identify them all. In such cases, the product name should be recorded and referred to the UMC, which will assist with identification. If the product is not already in the global WHO database, it will be added, together with the available information. A particular herbal product may have a number of indications and therefore appear in several places in the HATC classification.[10]

Local input by the reporter as to the precision or otherwise of the information on the product is most useful. This can be provided in free text, as a commentary on the report, or by the submission of the manufacturer’s information or the original packaging. A national inventory or catalog of medicinal plants may also serve as a reference on medicinal plants and their use in the community. In many countries, however, knowledge of medicinal plants and their medicinal use has not been documented. The establishment of a national inventory or catalog should therefore be encouraged.

If the finished herbal product concerned or its raw materials were imported from other countries, the drug regulatory authority of the exporting country may be able to provide helpful information.

The precise Latin binomial botanical name (genus, species, author; as well as name of family) of the medicinal plants concerned should be used whenever possible, together with information about the plant parts used and the extraction and preparation methods employed. This information allows accurate comparison with other reports. A common vernacular name may be used in order not to delay or cancel the submission of a report. National pharmacovigilance centers should collaborate with the pharmacognosy departments of universities and with botanists, zoologists, and botanical garden staff regarding taxonomic (botanical and chemical) identification and botanical and vernacular nomenclature.

### Data management[11]

*Data quality*: Strenuous efforts should be made to ensure that there are quality controls on data processing and that the data elements of reports are as complete and accurate as possible. Mechanisms to check for duplications should be instituted.

*Data storage*: Computer databases should be managed to as high a standard as possible to facilitate access to and use of the data. Software should be selected with expert advice so that analytic needs can be met.

*Data analysis*: Programs should be developed to provide for regular analyses and data output appropriate for local needs.

*Analysis of the global WHO database*: The global WHO database managed by the UMC is being improved on the basis of the proposed “Database management and classification for coding of herbal medicines,” of which the previously mentioned HATC is one part.[12] Data-mining techniques that have proved effective on the very large number of reports for other medicines will be used for signal detection on reports for herbal medicines. The success of these techniques depends on the volume and quality of data submitted by the national Pharmacovigilance centers.

*Support on technical and data management* is available from the WHO Collaborating Centre for International Drug Monitoring, UMC (http://www.who-umc.org/).

## CONCLUSION

Herbal medicines are widely used in health care in both developed and developing countries. However, in recent years, there have been several high-profile herbal safety concerns that have had an impact on the public health, and there is increasing recognition of the need to develop pharmacovigilance (safety monitoring) systems for herbal medicines. Pharmacovigilance for herbal medicines is, in many respects, in its infancy and monitoring the safety of herbal medicines presents unique challenges.

This meeting aims to provide a comprehensive and critical overview of the current state of pharmacovigilance activities for herbal medicines at the national and global levels. It will explore in depth the challenges that pharmacovigilance of herbal medicines presents, consider relevant emerging issues and what steps could and should be taken to improve safety monitoring for herbal medicines in the future.
